# Small Molecules Showing Significant Protection of Mice against Botulinum Neurotoxin Serotype A

**DOI:** 10.1371/journal.pone.0010129

**Published:** 2010-04-13

**Authors:** Yuan-Ping Pang, Jon Davis, Shaohua Wang, Jewn Giew Park, Madhusoodana P. Nambiar, James J. Schmidt, Charles B. Millard

**Affiliations:** 1 Computer-Aided Molecular Design Laboratory, Mayo Clinic, Rochester, Minnesota, United States of America; 2 Division of Biochemistry, Walter Reed Army Institute of Research, Silver Spring, Maryland, United States of America; 3 Integrated Toxicology Division, United States Army Medical Research Institute of Infectious Diseases, Fort Detrick, Maryland, United States of America; Charité-Universitätsmedizin Berlin, Germany

## Abstract

Botulinum neurotoxin serotype A (BoNTA) causes a life-threatening neuroparalytic disease known as botulism that could afflict large, unprotected populations if the toxin were employed in an act of bioterrorism. Current post-exposure therapy is limited to symptomatic treatment or passive immunization that is effective for treating infant botulism at a cost of US $45,300 per treatment regimen. Antibodies can neutralize the extracellular but not the intracellular BoNTA. Moreover, antibody production, storage, and administration in a mass casualty scenario pose logistical challenges. Alternatively, small-molecule inhibitors of BoNTA endopeptidase (BoNTAe) are sought to antagonize the extracellular or intracellular toxin. While several such molecules reportedly demonstrated efficacy in protecting cells against BoNTA, there is scant information to show that small molecules can significantly protect mammals against BoNTA. Herein we report the development of effective small-molecules BoNTAe inhibitors with promising *in vivo* pharmacokinetics. One such molecule has an *in vivo* half-life of 6.5 hours and is devoid of obvious sign of toxicity. Pre-treatment with this molecule at 2 mg/kg protected 100% and 70% of treated mice against BoNTA at 5 times of its median-lethal dose during the periods of 2 and 4 half-lives of the inhibitor, respectively. In contrast, 40% and 0% of untreated mice survived during the respective periods. Similar levels of protection were also observed with two other small molecules. These results demonstrate that small molecules can significantly protect mice against BoNTA and support the pursuit of small-molecule antagonists as a cost-effective alternative or as an adjunct to passive immunity for treating botulism.

## Introduction

Seven distinct serotypes (A to G) of the spore-forming *Clostridium botulinum* have been characterized based upon production of structurally and functionally unique botulinum neurotoxins (BoNTs) [1]. Such toxins can cause a life-threatening neuroparalytic disease known as botulism [1] by inhibiting normal release of the neurotransmitter acetylcholine at peripheral neuromuscular junctions and thereby causing prolonged flaccid paralysis, serious medical sequelae, or death [1]. Despite its toxicity, the purified and diluted BoNT serotype A (BoNTA) can be harnessed to treat cholinergic nerve and muscle dysfunctions, as well as for cosmetic treatment of facial wrinkles [2], [3]. Even in carefully controlled clinical scenarios, however, overdoses of BoNTA can occur and result in systemic botulism [4]; such incidents may rise as the number of therapeutic indications increases [5]. Mishaps also may occur involving the use of unregulated or counterfeit formulations of BoNTA at unknown concentrations [6]. Moreover, due to its long *in vivo* half-life (t_1/2_ >31 days [7]), BoNTA is a recognized biological weapon that has been sought or stockpiled by both small terrorist cells and large industrial countries [8], [9]. Recently, it has been projected that botulism could afflict a large number of unprotected civilians if a food supply, for example the milk production and distribution chain [10], were intentionally contaminated by the toxin in an act of bioterrorism. There is an urgent need for small-molecule BoNTA inhibitors as effective and safe post-exposure treatment for BoNTA intoxication to respond to food poisoning, accidental clinical overdoses, and mass-casualty situations.

Current post-exposure therapy is limited to symptomatic treatment or passive immunization that is effective for treating infant botulism [11] at a cost of US $45,300 per treatment regimen [12]. Antibodies can neutralize the extracellular but not the intracellular BoNTA. Moreover, antibody production, storage, and administration in a mass casualty scenario pose logistical challenges. To antagonize the extracellular or intracellular BoNTA, small molecules [13]–[20] have been developed to inhibit BoNTA endopeptidase (BoNTAe) – the catalytic domain of BoNTA that specifically cleaves a critical component of the neurosecretory apparatus required for acetylcholine release [21]. While several such molecules have demonstrated efficacy in protecting cells against BoNTA [13], [15], [20], there is scant information to show that small molecules can significantly protect mammals against BoNTA, although an *in vivo* study of small-molecule BoNTAe inhibitors has been reported [22].

Herein, we report the development of effective small-molecule BoNTAe inhibitors with *in vivo* half-live of 4–6 hours. These inhibitors showed 100% and 70% of protection of mice against BoNTA at 5 times of its median-lethal dose during the periods of 2 and 4 half-lives of the inhibitors at an inhibitor concentration of 2 mg/kg, respectively. We also discuss the prospect of small-molecule inhibitors as a cost-effective alternative or as an adjunct to passive immunity for treating botulism.

## Results

### Design and Synthesis

**Figure 1:**
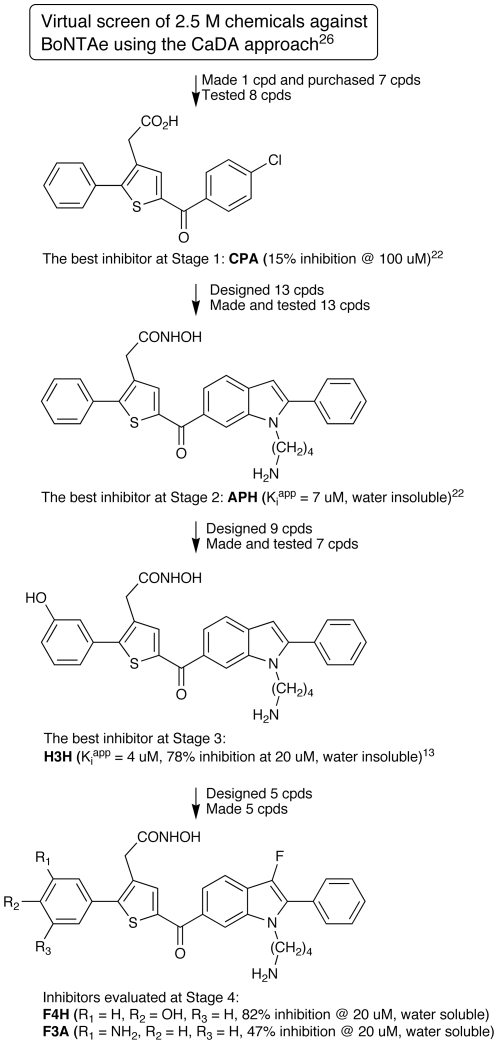
The development process of H3H, F4H and F3A as small-molecule BoNTAe inhibitors.

We previously reported a serotype-specific, small-molecule BoNTAe inhibitor, **H3H** (structure shown in Figure 1), which has a *K*_i_^app^ value of 3.8±0.77 µM and was resulted from our lead identification and optimization as summarized in Figure 1 [14], [23]. One drawback of **H3H** is insolubility in water. In optimizing **H3H** for water solubility and higher potency in inhibiting BoNTAe, we encountered problems in derivatizing **H3H** caused by chemical instability under acidic conditions (pH<2.0) that was presumably due to the proton at position 3 of the indole ring. These problems hampered the structural modifications of **H3H** guided by insights from computer simulations or the crystal structures of inhibitor-bound BoNTAe complexes.

Recognizing the synthesis step as the rate-determining step of the optimization, we set out to first establish a facile synthetic scheme that can lead to a group of inhibitor analogues and then use computer simulations of the inhibitor-bound BoNTAe complexes to prioritize the syntheses of the analogues. This was different from what we did earlier, namely, first finding alternative analogues on the basis of computer simulations and then determining whether the alternatives were synthetically accessible.

**Figure 2:**
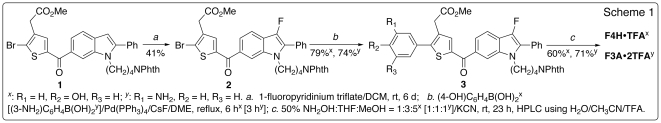
Synthetic scheme for F4H and F3A.

Accordingly, we developed a simple synthetic scheme shown Figure 2 that begins with a known intermediate used for the synthesis of **H3H** [14]. The new scheme, which readily leads to a handful of new analogues of **H3H** by varying substituents R_1_, R_2_, and R_3_, enabled us to address the problems of water solubility and chemical instability of **H3H** by introducing hydrophilic groups and replacing the position-3 proton of the indole ring with a fluorine atom [24], respectively. Preliminary multiple molecular dynamics simulations (10 1-ns-long simulations) suggested that two of such analogues, **F3A** and **F4H** (structures shown in Figure 1), might be able to interact favourably with the active site of BoNTAe. The simulation results were later supported by the extended multiple molecular dynamics simulations (10 10-ns-long simulations) described below.

Therefore, we made **F4H** and **F3A** with relative ease according to the scheme shown in Figure 2. Gratifyingly, we found that both **F4H** and **F3A** are water soluble at concentrations up to 5.0 mM and stable under acidic conditions.

### Computer Simulation

**Figure 3:**
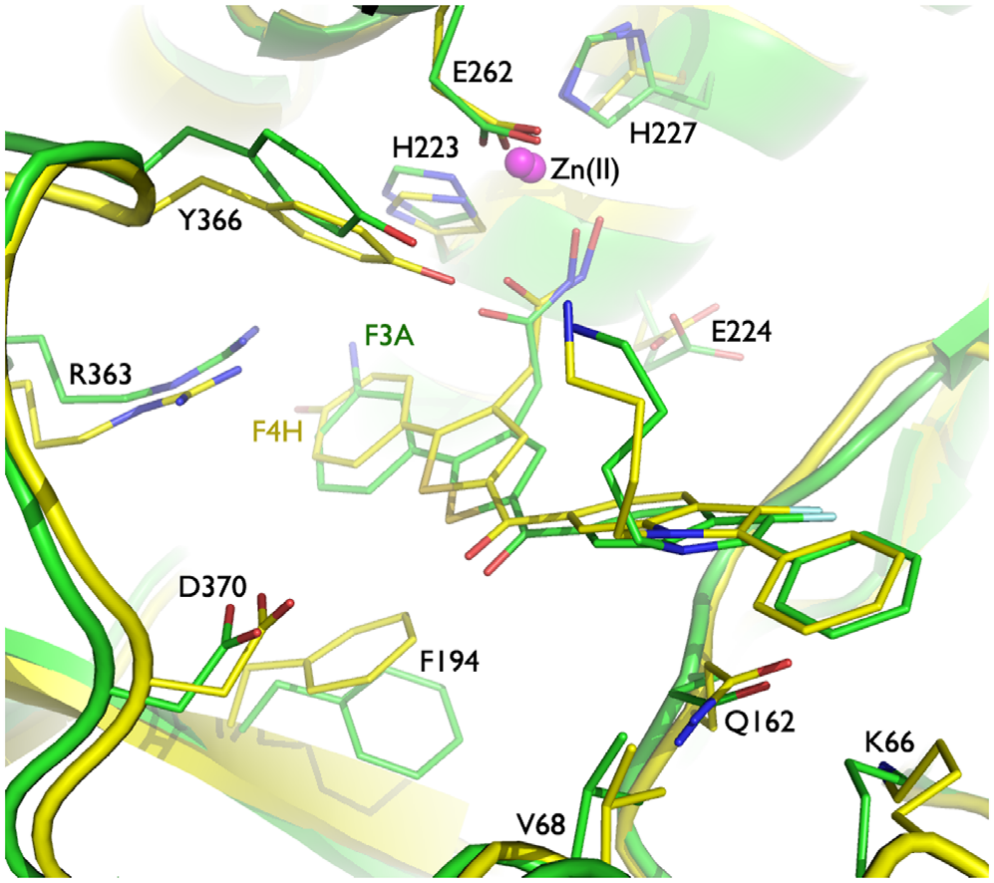
Overlay of simulation-generated models of F4H•BoNTAe (yellow) and F3A•BoNTAe (green). For clarity the water molecules that bridge the interaction between Asp370 and the ketone oxygen atom are not displayed, but these water molecules along with other active-site water molecules are included in the coordinates of Datasets S1 and S2.

Subsequent extended multiple molecular dynamics simulations (10 10-ns-long simulations) of BoNTAe in complex with **F4H** or **F3A** suggested that both inhibitors have (1) the hydroxamate coordinating the zinc ion embedded in the active site, (2) the hydroxamate forming a hydrogen bond to Glu224, (3) the cation-pi interaction of the thiophene-substituted phenyl group with Arg363, (4) the pi-pi interactions of the thiophene-substituted phenyl group with Phe194 and Tyr366, (5) the interaction of the ketone oxygen atom with Asp370 that is bridged by at least one water molecule, and (6) the cation-pi and pi-pi interactions of the indole-substituted phenyl group with Lys66 and Gln162, respectively (Figure 3). The main differences between the two inhibitor complexes are that (1) the thiophene-substituted phenyl group has stronger pi-pi interactions (judged by distance) with Tyr366 and Phe194 in **F4H**•BoNTAe than in **F3A**•BoNTAe, (2) Tyr366 forms a hydrogen bond with the carbonyl oxygen atom of the hydroxamate in **F4H**•BoNTAe but not in **F3A**•BoNTAe, and (3) the interaction between the ketone oxygen atom and Asp370 is bridged by one or two water molecules in **F4H**•BoNTAe or **F3A**•BoNTAe, respectively. The coordinates of the simulation-generated **F4H**•BoNTAe and **F3A**•BoNTAe complexes are available in Datasets S1 and S2, respectively.

### Biological Evaluation

High performance liquid chromatography (HPLC)-based BoNTAe inhibition assays [25] showed that **F4H** is as potent as **H3H** in inhibiting BoNTAe, and **F3A** is less potent than **H3H** (Table 1). Furthermore, **H3H**, **F4H**, and **F3A** showed no acute toxicity to mice. We therefore performed *in vivo* pharmacokinetic studies on all three inhibitors. Interestingly, the exposures of **F4H** and **F3A** to mice are nearly the same but slightly less than that of **H3H**, as measured by the area under the time-concentration curve (AUC), even though the maximum concentration (C_max_) and the concentration 24 hours after one dose of a test compound (C_24_) for each inhibitor are different (Table 1). The nearly identical half-lives (t_1/2_≈6 hours) of **F4H** and **F3A** are longer than that of **H3H** (t_1/2_≈4 hours). In this context, we further evaluated all three inhibitors using a standardized mouse model of botulism [26] to determine if they can protect mice against either extracellular or intracellular BoNTA during the period of 8 half-lives of the test inhibitor in a single-dose experiment.

**Table 1:** *In Vitro* Inhibition of BoNTAe and *in Vivo* Pharmacokinetic Data for H3H, F4H, and F3A.

Inhibitor	% BoNTAe inhibition^1^	C_max_ (ng/mL)	C_24_ (ng/mL)	AUC_last_ (hr•ng/mL)	T_1/2_ (hr)
**H3H**	78±4	497.4	3.0	1547.3	4.35
**F4H**	82±6	738.4	<0.5	1386.4	6.50
**F3A**	47±1	256.0	7.0	1385.9	6.25

1 The inhibition assays were conducted at an inhibitor concentration of 20 µM.

**Figure 4:**
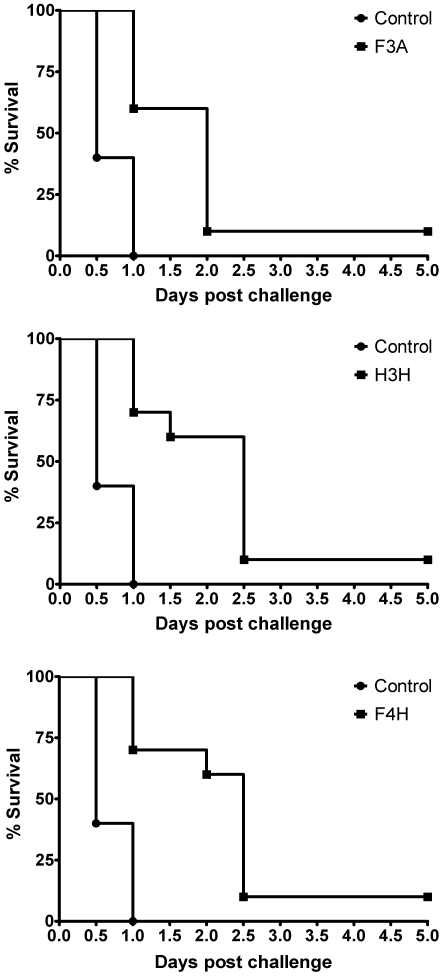
The survival curves of mice treated with placebo or a BoNTAe inhibitor. **F3A**: top, **H3H**: middle, and **F4H**: bottom.

Groups of Balb/c mice were given one 2-mg/kg intraperitoneal injection of **H3H**, **F4H**, **F3A**, or dimethyl sulfoxide as a control and, after 30 minutes, each mouse was challenged intraperitoneally with BoNTA at 5 times of its median-lethal dose. All mice were examined twice daily for survival, behaviour, motor activity, breath, and extraocular symptoms of botulism. Each of the three inhibitors significantly (p<0.05) increased survival at different time intervals (Figure 4). Importantly, all mice treated with any of the three inhibitors survived during the 12-hour period (∼2t_1/2_ for **F4H**) after the BoNTA challenge. During this period, the inhibitors are expected to work optimally according to the time course of the inhibitor concentration in mouse plasma. In contrast, 60% of the untreated mice died during the 12-hour period. Consistently, all untreated mice died 24 hours (∼4t_1/2_ for **F4H**) after the challenge, whereas 70% and 60% of the **F4H**-treated mice survived 24 hours and 48 hours (∼8t_1/2_ for **F4H**) after the challenge, respectively (Figure 4). Furthermore, 10% of the mice treated with any of the three inhibitors survived without symptoms of botulism until they were euthanized on day 5 (Figure 4).

## Discussion

Small-molecule BoNTAe inhibitors have been pursued actively by different research groups [13]–[20], but concern remains with regard to the feasibility of the small-molecule therapy for botulism, primarily because (1) BoNTA has a long *in vivo* half-life (t_1/2_ >31 days [7]), (2) small-molecule BoNTAe inhibitors with low nanomolar potencies are difficult to obtain [19], and (3) there has been only one article to date reporting an *in vivo* study of small-molecule BoNTAe inhibitors [22]. The work described above offers the following insights into the prospect of the small-molecule botulism therapy, although additional studies are needed to determine if the observed protection of mice against BoNTA by the pre-treatment of **F4H**, **H3H**, or **F3A** involves inhibition and clearance of extracellular toxin depots, uptake by intoxicated neurons, or both routes.

**F4H**, **H3H**, and **F3A** have *in vivo* half-lives of 4–6 hours, and all mice treated with any of the three inhibitors survived during the 12-hour period (∼2t_1/2_ for **F4H**) after the BoNTA challenge. It is therefore plausible that the problem with a long *in vivo* half-life of BoNTA can be mitigated by treating with an **F4H**-like compound one dose per day for multiple days. This treatment could be shortened if the compound were used in combination with long-lasting antibodies [27] that are effective to neutralize the extracellular toxin.

**F4H** showed 82±6% inhibition of BoNTAe at the inhibitor concentration of 20 µM. However, with one 2-mg/kg intraperitoneal injection, **F4H** showed 100, 70, and 60% protection of mice against BoNTA during the 12-, 24-, and 48-hour periods after the toxin challenge, respectively. This suggests that small-molecule BoNTAe inhibitors with low nanomolar potencies might not be necessary; inhibitors with low micromolar or high nanomolar potencies may suffice.

All three different inhibitors protected 100% of treated mice during the 12-hour period (∼2t_1/2_ for **F4H**) and 10% of the mice during the standard 5-day observation period, with a single intraperitoneal injection of the inhibitor against a supralethal BoNTA challenge. Furthermore, 90% of the **F3A**-treated mice, 40% of the **H3H**-treated mice, and 40% of the **F4H**-treated mice died 48 hours after the toxin challenge, respectively (Figure 4). The *in vivo* potencies appeared to be consistent with the *in vitro* potencies of the three inhibitors (Table 1). These results support the hypothesis that protection of mice against BoNTA can be achieved by treatment with a small-molecule BoNTAe inhibitor and are incentive to improve BoNTAe inhibitor structures and dosing regimen to optimize *in vivo* efficacies.

In summary, the present work demonstrates that small-molecule inhibitors can significantly protect mice against BoNTA and encourages the pursuit of small-molecule BoNTAe inhibitors for alternative or complementary treatment of botulism.

## Materials and Methods

The animal experiments were performed with an approved protocol by the Institutional Animal Care and Use Committee at the Walter Reed Army of Institute of Research (IACUC number: B02-08) that is in compliance with the Animal Welfare Act and other United States federal statutes and regulations involving animals and adheres to principles stated in the Guide for the Care and Use of Laboratory Animals, NRC Publication, 1996 edition.

### Reagents

Hexanes (Hex), ethyl acetate (EtOAc), and trifluoroacetic acid (TFA) were purchased from Fisher Scientific (Pittsburgh, PA). BSA, HEPES buffer, and zinc chloride were purchased from Sigma-Aldrich (St. Louis, MO). Dithiothreitol was obtained from BioRad (Hercules, CA). All commercially available reagents were used as received. Recombinant BoNTAe was provided by Dr. Leonard Smith of the United States Army Medical Research Institute of Infectious Diseases, Fort Detrick, MD.

### Chemical Synthesis

#### General Description

The ^1^H NMR (400 MHz) and ^13^C NMR (100 MHz) spectra were recorded on a Mercury 400 spectrometer from Varian (Palo Alto, CA). Chemical shifts are reported in ppm using either tetramethylsilane or the solvent peak as an internal standard. Data are reported as follows: chemical shift, multiplicity (s  =  singlet, brs  =  broad singlet, d  =  doublet, t  =  triplet, brt  =  broad triplet, q  =  quartet, m  =  multiplet), coupling constant, and integration. Low-resolution mass spectra were recorded using either Hewlet Packard 5973 Mass Spectrometer with SIS Direct Insertion Probe (Palo Alto, CA) or Waters ZQ/EMD 1000 Mass Spectrometer (Milford, MA). High-resolution mass spectra were obtained on a Bruker BioTOF II ESI. IR spectra were obtained on a ThermoNicolet Avatar 370 FT-IR (Waltham, MA) using KBr pellet. Medium pressure liquid chromatography (MPLC) was performed with Biotage SP-1 (Charlottesville, VA) using silica gel (EM Science, 230–400 mesh). HPLC was carried out on a 5-µm C18 column (analytical: 4.60×250 mm, HyperClone; semi-preparative: 21.2×250 mm, Gemini) from Phenomenex (Torrance, CA) eluting with linear gradient of 80% of solution A (1000 mL of H_2_O and 1 mL of TFA) to 100% of solution B (100 mL of H_2_O, 900 mL of MeCN and 1 mL of TFA) over 20 minutes at a flow rate of 1.0 mL/min (analytical) or over a specified amount of time at a flow rate of 10 mL/min (semi-preparative) with UV detection at 254 nm on a Beckman Coulter System Gold HPLC system (166P detector and 125P solvent module) from Beckman Coulter (Brea, CA). KCN is highly toxic and must be handled with extreme care by trained personnel.

#### Methyl 2-(2-bromo-5-(1-(4-(1,3-dioxoisoindolin-2-yl)butyl)-3-fluoro-2-phenyl-1*H*-indole-6-carbonyl)thiophen-3-yl)acetate (2)

To a solution of methyl 2-(2-bromo-5-(1-(4-(1,3-dioxoisoindolin-2-yl)butyl)-2-phenyl-1*H*-indole-6-carbonyl)-thiophen-3-yl)acetate (**1** in Figure 2) [14] (156 mg, 0.24 mmol) in 3 mL CH_2_Cl_2_ was added 1-fluoropyridinium triflate (78 mg, 0.28 mmol), and then the mixture was stirred at room temperature for 6 days. The resulting mixture was diluted with 40 mL Et_2_O, washed with brine (2×10 mL), dried over MgSO_4_, filtered, and then concentrated *in vacuo*. MPLC purification (Hex∶EtOAc/9∶1) gave **2** (66 mg, 41%) as a yellow solid foam. ^1^H NMR (CDCl_3_) δ 7.94 (s, 1H), 7.78−7.75 (m, 2H), 7.71−7.63 (m, 4 H), 7.57 (s, 1H), 7.50−7.39 (m, 5H), 4.25 (t, *J* = 7.2 Hz, 2H), 3.71 (s, 3H), 3.69 (s, 2H), 3.49 (t, *J* = 6.6 Hz, 2H), 1.67−1.60 (m, 2H), and 1.50−1.43 (m, 2H) (see Figure S1 for proton NMR spectrum of **2**); ^13^C NMR (CDCl_3_) δ 187.42, 170.49, 168.57, 143.91, 141.87 (^1^ *J*_CF_ = 244.0 Hz), 136.06, 135.98, 135.02, 134.26, 132.30 (^3^ *J*_CF_ = 7.0 Hz), 132.08, 131.54, 129.95, 129.17, 128.44 (^3^ *J*_CF_ = 3.0 Hz), 127.60 (^2^ *J*_CF_ = 21.0 Hz), 123.48, 122.10, 122.20, 120.17 (^2^ *J*_CF_ = 16.0 Hz), 117.04 (^3^ *J*_CF_ = 3.0 Hz), 112.66, 52.61 (q, *J* = 10.7 Hz), 43.64, 37.23, 35.07, 27.32, and 25.76; IR cm^−1^ 2921.2, 1707.6, and 1393.0; LRMS-EI m/z 672 and 674 (12% each, [M^+^]), 160 (100%, [CH_2_NPhth^+^]); HRMS-ESI calculated for C_34_H_26_BrFN_2_O_5_SNa^+^ [M+Na^+^] 695.0622, found 695.0619.

#### Methyl 2-(5-(1-(4-(1,3-dioxoisoindolin-2-yl)butyl)-3-fluoro-2-phenyl-1*H*-indole-6-carbonyl)-2-(4-hydroxyphenyl)thiophen-3-yl) (3x)

A mixture of **2** (42 mg, 0.062 mmol), Pd(PPh_3_)_4_ (8 mg, 0.007 mmol), CsF (28 mg, 0.18 mmol), 4-hydroxyphenylboronic acid (13 mg, 0.094 mmol), and H_2_O (200 µL) in 1,2-dimethoxyethane (8 mL) was degassed with N_2_ for 10 minutes and then refluxed for 6 hours. The resulting suspension was poured into H_2_O (10 mL) and then extracted with 70 mL Et_2_O. The organic layer was washed with brine (2×10 mL), dried over MgSO_4_, and then concentrated *in vacuo*. MPLC purification (Hex∶EtOAc/5∶1) of the residue gave **3x** as a yellow solid foam (34 mg, 79%). ^1^H NMR (CDCl_3_) δ 7.99 (s, 1H), 7.78−7.66 (m, 7H), 7.50−7.38 (m, 7H), 6.94 (d, *J* = 8.4 Hz, 2H), 6.27 (s, 1H), 4.26 (t, *J* = 7.0 Hz, 2H), 3.69 (m, 5H), 3.48 (t, *J* = 6.8 Hz, 2H), 1.68−1.59 (m, 2H), and 1.50−1.43 (m, 2H) (see Figure S2 for proton NMR spectrum of **3x**); ^13^C NMR (CDCl_3_) δ 188.36, 171.87, 168.57, 157.05, 149.86, 141.93 (^1^ *J*_CF_ = 244.6 Hz), 141.28, 137.87, 137.80, 134.24, 132.38, 132.35, 132.10, 131.03, 130.98 (^3^ *J*_CF_ = 3.0 Hz), 129.98, 129.16, 128.56 (^3^ *J*_CF_ = 3.0 Hz), 127.28 (^2^ *J*_CF_ = 15.3 Hz), 125.25, 123.49, 121.42, 119.98 (^2^ *J*_CF_ = 15.3 Hz), 116.93, 116.17, 112.64, 52.53 (q, *J* = 9.9 Hz), 43.61, 37.27, 34.49, 27.35, and 25.77; IR cm^−1^ 3391.2, 2929.4, 2851.8, 1711.7, and 1442.0; LRMS-EI m/z 687 (100%, [M^+^]), 439 (65%); HRMS-ESI calculated for C_40_H_31_FN_2_O_6_SNa^+^ [M+Na^+^] 709.1779, found 709.1787.

#### 2-(5-(1-(4-Aminobutyl)-3-fluoro-2-phenyl-1H-indole-6-carbonyl)-2-(4-hydroxyphenyl)thiophen-3-yl)-*N*-hydroxyacetamide(F4H)

To a stirred solution of **3x** (34 mg, 0.049 mmol) in THF/MeOH (3 mL/5 mL), 1 mL of 50% aqueous NH_2_OH was added, followed by a catalytic amount (two crystals) of KCN. The resulting mixture was stirred for 23 hours at room temperature, and then filtered through a short Celite column. HPLC purification of the filtrate gave **F4H**•**TFA** as a yellow amorphous solid (20 mg, 60%). The semi-preparative and analytical HPLC retention times of **F4H**•**TFA** are 14.00 and 14.57 minutes, respectively (see Figure S3 for chromatograms of **F4H**•**TFA** before and after the HPLC purification). ^1^H NMR (CD_3_OD) δ 8.11 (s, 1H), 7.78 (s, 1H), 7.73 (d, *J* = 8.4 Hz, 1H), 7.66 (dd, *J* = 1.2, 8.4 Hz, 1H), 7.59−7.58 (m, 4H), 7.54−7.50 (m, 1H), 7.39 (d, *J* = 8.6 Hz, 2H), 6.92 (d, *J* = 8.6 Hz, 2H), 4.36 (t, *J* = 7.2 Hz, 2H), 3.51 (s, 2H), 2.79 (t, *J* = 7.2 Hz, 2H), 1.76−1.69 (m, 2H), and 1.50−1.43 (m, 2H) (see Figure S4 for proton NMR spectrum of **F4H**); ^13^C NMR (CD_3_OD) δ 188.80, 169.26, 160.90 (q, CF_3_ CO_2_H, ^2^ *J*_CF_ = 36.6 Hz), 158.74, 150.11, 141.75 (^1^ *J*_CF_ = 243.1 Hz), 140.72, 137.85, 132.22, 132.15 (^3^ *J*_CF_ = 5.3 Hz), 130.73, 130.53, 129.85, 129.02, 128.96, 128.45 (^3^ *J*_CF_ = 3.8 Hz), 127.30 (^2^ *J*_CF_ = 20.6 Hz), 123.86, 120.58, 119.64 (^2^ *J*_CF_ = 16.0 Hz), 116.65, 115.71, 113.10, 43.29, 39.18, 32.29, 27.11, and 24.70; IR cm^−1^ 3438.5, 3227.7, 1677.0, 1608.9, 1551.6, 1428.1, 1250.1, 1202.9, 1138.2; LRMS-EI m/z 558 (48%, [M^+^]), 309 (36%); HRMS-ESI calculated for C_31_H_29_FN_3_O_4_S^+^ [M+H^+^] 558.1857, found 558.1901.

#### Methyl 2-(2-(3-aminophenyl)-5-(1-(4-(1,3-dioxoisoindolin-2-yl)butyl)-3-fluoro-2-phenyl-1*H*-indole-6-carbonyl)thiophen-3-yl)acetate (3y)

A mixture of **2** (20 mg, 0.03 mmol), Pd(PPh_3_)_4_ (7 mg, 0.006 mmol), CsF (13 mg, 0.09 mmol), 3-aminophenylboronic acid (6 mg, 0.04 mmol), and H_2_O (60 µL) in 1,2-dimethoxyethane (4 mL) was degassed with N_2_ for 10 minutes and then refluxed until all the starting ester had been consumed (3 hours). The resulting black suspension was poured into H_2_O (10 mL) and then extracted with 40 mL Et_2_O. The organic layer was washed with brine (2×20 mL), dried over MgSO_4_, and then concentrated *in vacuo*. MPLC purification (Hex∶EtOAc/5∶1) of the residue gave **3y** as a yellow solid foam (15 mg, 74%). ^1^H NMR (CDCl_3_) δ 7.99 (s, 1H), 7.78−7.76 (m, 2H), 7.71−7.67 (m, 5H), 7.51−7.37 (m, 5H), 7.24 (t, *J* = 7.6 Hz, 1H), 6.88 (d, *J* = 7.2 Hz, 1H), 6.83 (s, 1H), 6.73 (d, *J* = 7.2 Hz, 1H), 4.26 (t, *J* = 7.0 Hz, 2H), 3.72 (s, 2H), 3.69 (s, 3H), 3.48 (t, *J* = 6.8 Hz, 2H), 1.66−1.58 (m, 2H), and 1.50−1.45 (m, 2H) (see Figure S5 for proton NMR spectrum of **3y**); ^13^C NMR (CDCl_3_) δ 188.12, 171.73, 168.51, 149.72, 147.07, 141.92 (^1^ *J*_CF_ = 245.0 Hz), 141.74, 137.49, 134.19, 134.09, 132.46, 132.36 (^3^ *J*_CF_ = 6.0 Hz), 132.13, 130.36, 129.96, 129.15, 129.05, 128.58 (^3^ *J*_CF_ = 3.0 Hz), 127.21 (^2^ *J*_CF_ = 10.7 Hz), 123.46, 121.39, 119.94 (^3^ *J*_CF_ = 6.1 Hz), 119.71, 116.89, 115.80 (^2^ *J*_CF_ = 15.3 Hz), 112.59, 52.41, 43.61, 37.25, 34.50, 27.36, and 25.77; IR cm^−1^ 3456.5, 3366.6, 2945.7, 1711.7, 1601.4 and 1393.0; LRMS-EI m/z 686 (100%, [M^+^]); HRMS-ESI calculated for C_40_H_33_FN_3_O_5_S^+^ [M+H^+^] 686.2119, found 686.2128.

#### 2-(5-(1-(4-Aminobutyl)-3-fluoro-2-phenyl-1*H*-indole-6-carbonyl)-2-(3-aminophenyl)thiophen-3-yl)-*N*-hydroxyacetamide (F3A)

To a stirred solution of **3y** (15 mg, 0.022 mmol) in THF/MeOH (0.5 mL/0.5 mL), 0.5 mL of 50% aqueous NH_2_OH was added, followed by a catalytic amount (two crystals) of KCN. The resulting mixture was stirred for 16 hours at room temperature, and then filtered through a short Celite column. HPLC purification (eluting time: 20 minutes) of the filtrate gave **F3A**•**2TFA** as a yellow solid foam (12 mg, 71%). The semi-preparative and analytical HPLC retention times of **F3A**•**2TFA** are 13.12 and 12.25 minutes, respectively (see Figure S6 for chromatograms of **F3A**•**2TFA** before and after the HPLC purification). ^1^H NMR (CD_3_OD) δ 8.12 (s, 1H), 7.82 (s, 1H), 7.74 (d, *J* = 8.4 Hz, 1H), 7.69 (d, *J* = 8.4 Hz, 1H), 7.66−7.51 (m, 8H), 7.44−7.42 (m, 1H), 4.37 (t, *J* = 6.8 Hz, 2H), 3.54 (s, 2H), 2.78 (t, *J* = 7.2 Hz, 2H), 1.76−1.68 (m, 2H), and 1.49−1.41 (m, 2H) (see Figure S7 for proton NMR spectrum of **F3A**); ^13^C NMR (CD_3_OD) δ 188.54, 168.75, 160.22 (q, CF_3_ CO_2_H, ^2^ *J*_CF_ = 39.7 Hz), 146.93, 142.57, 141.73 (^1^ *J*_CF_ = 243.1 Hz), 137.34, 134.89, 134.07, 132.54, 132.20 (^3^ *J*_CF_ = 5.4 Hz), 131.88, 130.72, 129.86, 129.08, 128.99, 128.38 (^3^ *J*_CF_ = 3.1 Hz), 128.29, 127.57 (^2^ *J*_CF_ = 20.5 Hz), 122.62 (^2^ *J*_CF_ = 32.0 Hz), 120.69, 119.83 (^2^ *J*_CF_ = 15.3 Hz), 116.65, 116.23 (q, CF_3_CO_2_H, ^1^ *J*_CF_ = 287.4 Hz), 113.08, 43.28, 39.15, 32.28, 27.06, and 24.67; IR cm^−1^ 3432.0, 2925.3, 1679.0, 1200.9, 1135.5; LRMS-EI m/z 557 (60%, [M^+^]), 309 (62%); HRMS-ESI calculated for C_31_H_30_FN_4_O_3_S^+^ [M+H^+^] 557.2017, found 557.2040.

### *in Vitro* Evaluation

Assays of the BoNTAe activity were done at 37°C and contained 0.5 mM substrate, 0.5–1.5 µg/mL recombinant BoNTAe, 40 mM HEPES, 1 mM dithiothreitol, 25 µM ZnCl_2_, 0.5 mg/mL BSA, and 0.05% tween at pH 7.3. Substrate for BoNTAe was an SNAP-25 fragment containing residues 187–203 with *N*- and *C*-termini acylated and amidated, respectively [28]. Inhibitors were dissolved in dimethyl sulfoxide at 10 times the final assay concentration, then diluted into the assay mixture containing substrate, followed by addition of the endopeptidase (*i.e.*, inhibitor and endopeptidase were not preincubated). Assay times and endopeptidase concentrations were adjusted so that less than 10% of the substrate was hydrolyzed. Assays were stopped by acidification with TFA and analyzed by reverse-phase HPLC as described previously [25].

### *in Vivo* Evaluation

#### Pharmacokinetics Study

The *in vivo* pharmacokinetic parameters were determined by dosing 6 Balb/c mice intraperitoneally with a test inhibitor at 2 mg/kg at which concentration no obvious sign of toxicity was observed. Blood was collected by cardiac puncture at 0.5, 1, 2, 4, 8, and 24 hours and the plasma was separated and kept frozen at −80°C until processing. Each experiment was repeated three times. The plasma was thawed and extracted with two volumes of ice-cold acetonitrile to precipitate plasma proteins and release the inhibitor. The organic phase was analyzed by liquid chromatography mass spectrometry and the concentration of the inhibitor was determined based on a standard curve run in parallel. The stability of the inhibitor in acetonitrile was determined prior to analyzing pharmacokinetic samples. Pharmacokinetic values were determined with WinNonLin software from Pharcite based on the plasma concentration curve.

#### Protection Study

The protection studies were carried out by using a standardized mouse model of botulism [26]. Briefly, groups of Balb/c mice were given a single 2-mg/kg intraperitoneal injection of **H3H**, **F4H**, **F3A** or dimethyl sulfoxide as a control and, after 30 minutes, each mouse was challenged intraperitoneally with BoNTA at 5 times of its median-lethal dose. Dimethyl sulfoxide was used as a carrier vehicle because **H3H** is water insoluble. All mice were examined twice daily for survival, behaviour, motor activity, breath, and extraocular symptoms of botulism. The numbers of mice in the treated and control groups were 10 and 5, respectively. Survival curves were constructed based on the number of survivors and statistically analyzed using GraphPad Prism 5.0 (Graphpad Software, Inc.).

### Computer Simulations

#### Model Preparation

The atomic charges of **F4H** and **F3A** were obtained according to the RESP procedure [29] with *ab initio* calculations at the HF/6-31G*//HF/6-31G* level using the Gaussian 98 program [30]. The starting structure of inhibitor•BoNTAe was generated by (1) manually docking the inhibitor into the BoNTAe active site and (2) replacing the active-site zinc ion with the tetrahedral zinc ion using the cationic dummy atom approach [23], [31]–[33]. In the manual docking, the hydroxamate group was placed near the tetrahedral zinc ion, the thiophene-substituted phenyl group was placed near Arg363, and the ammonium group was placed near Glu64. The BoNTAe structure used for the docking was taken from the crystal structure of an inhibitor-bound BoNTAe (Protein Data Bank Code: 3BOO [34]) whose conformations of missing residues 62–67 were taken from the crystal structure of a BoNTAe mutant in complex with SNAP-25 (Protein Data Bank Code: 1XTG [35]). For BoNTAe, His223 and His227 were treated as HIN (histidinate) [32], [36], [37]; His39, His230, and His269 were treated as HID; all other His residues were treated as HIP; Glu261 and Glu351 were treated as GLH [32], [36], [37]. A total of 111 crystallographically determined water molecules (named HOH) located inside the enzyme were included for simulations. The topology and coordinate files of the water-containing inhibitor•BoNTAe complex were generated by the PREP, LINK, EDIT, and PARM modules of the AMBER 5.0 program [38]. The complex was refined by energy minimization using a dielectric constant of 1.0 and 100 cycles of steepest-descent minimization followed by 100 cycles of conjugate-gradient minimization. The refined complex was solvated with 13,617 and 13,540 TIP3P water molecules (named WAT) [39] for **F4H** and **F3A**, leading to a system of 48,096 and 47,866 atoms, respectively. The WAT molecules were obtained from solvating the complex using a pre-equilibrated box of 216,000 TIP3P molecules, whose hydrogen atom charge was set to 0.4170, where any water molecule was removed if it had an oxygen atom closer than 2.2 Å to any solute atom or a hydrogen atom closer than 2.0 Å to any solute atom, or if it was located further than 9.0 Å along the x-, y-, or z-axis from any solute atom.

#### Multiple Molecular Dynamics Simulations

The solvated complex system was energy-minimized for 100 cycles of steepest-descent minimization followed by 100 cycles of conjugate-gradient minimization to remove close van der Waals contacts in the system, then heated from 0 to 300 K at a rate of 10 K/ps under constant temperature and volume, and finally simulated independently with a unique seed number for initial velocities at 300 K under constant temperature and pressure using the PMEMD module of the AMBER 8.0 program [40] with the AMBER force field (ff99SB) [41], [42]. All simulations used (1) a dielectric constant of 1.0, (2) the Berendsen coupling algorithm [43], (3) a periodic boundary condition at a constant temperature of 300 K and a constant pressure of 1 atm with isotropic molecule-based scaling, (4) the Particle Mesh Ewald method to calculate long-range electrostatic interactions [44], (5) a time step of 1.0 fs, (6) the SHAKE-bond-length constraints applied to all the bonds involving the H atom, (7) saving the image closest to the middle of the “primary box” to the restart and trajectory files, (8) formatted restart file, and (9) default values of all other inputs of the PMEMD module. Ten different molecular dynamics simulations (each lasted 10 ns) were carried out for the BoNTAe in complex with **F4H** or **F3A** on a cluster of Apple Mac Pros with 80 Intel Xeon cores (3.0 GHz).

#### Simulation Analysis

For each of the 10 simulations of **F4H**•BoNTAe or **F3A**•BoNTAe, 100 instantaneous conformations were saved at 10-ps intervals during the last 1-ns period. A total of 1,000 instantaneous conformations of **F4H**•BoNTAe or **F3A**•BoNTAe from the 10 simulations were subjected to a cluster analysis using the averagelinkage algorithm (epsilon = 2.0 Å and RMS on alpha-carbon atoms) [45] implemented in the PTRAJ module of the AMBER 10 program [40]. Only one cluster of the BoNTAe conformations was identified. All 1,000 instantaneous conformations of **F4H**•BoNTAe or **F3A**•BoNTAe were subjected to a second-round cluster analysis using the averagelinkage algorithm (epsilon = 1.5 Å and RMS on all atoms of **F4H or F3A**) [45]. This analysis identified 7 and 4 clusters for the **F4H** and **F3A** conformations, respectively. The numbers of the **F4H** conformations in Clusters 1–7 are 200, 100, 423, 27, 150, 30, and 70, respectively; the numbers of the **F3A** conformations in Clusters 1–4 are 600, 299, 1, and 100, respectively. The representative conformations of **F4H**•BoNTAe and **F3A**•BoNTAe from their most populated clusters overlay reasonably well (see Figure 3) and are considered as plausible complex structures in water. The coordinates of the representative conformations are available from Datasets S1 and S2. The coordinates of other conformations are available upon request.

## Supporting Information

Figure S1 Proton NMR spectrum of 2.
(0.79 MB PDF)

Figure S2 Proton NMR spectrum of 3x.
(0.56 MB PDF)

Figure S3 Chromatograms of F4H⋅TFA before and after the HPLC purification.
(0.17 MB PDF)

Figure S4 Proton NMR spectrum of F4H.
(0.54 MB PDF)

Figure S5 Proton NMR spectrum of 3y.
(0.50 MB PDF)

Figure S6 Chromatograms of F3A⋅2TFA before and after the HPLC purification.
(0.17 MB PDF)

Figure S7 Proton NMR spectrum of F3A.
(0.43 MB PDF)

Dataset S1 Coordinates of simulation-generated model of F4H⋅BoNTAe.
(0.46 MB TXT)

Dataset S2 Coordinates of simulation-generated model of F3A⋅BoNTAe.
(0.47 MB TXT)
