# Supplementary material for: Small Molecules Showing Significant Protection of Mice against Botulinum Neurotoxin Serotype A
Source: PLoS One. 2010 Apr 13;5(4):e10129. doi: 10.1371/journal.pone.0010129 (PMC2854131; doi:10.1371/journal.pone.0010129)

```
jgp18101-2

exp1  s2pul

      SAMPLE                SPECIAL
date   Feb 20 2010  temp        not used
solvent      CDCl3  gain        not used
file /export/home/~ spin              20
pang/stephen/jgp18~ hst            0.008
  /jgp18101-2.fid   pw90          14.900
  ACQUISITION       alfa          20.000
sw          6398.0            FLAGS
at           3.002  il                 n
np           38412  in                 n
fb        not used  dp                 y
bs              16  hs                nn
d1           1.000          PROCESSING
nt               8  lb              0.50
ct               8  fn          not used
  TRANSMITTER                DISPLAY
tn              H1  sp            -798.3
sfrq       400.088  wp            6397.8
tof          417.3  rfl            798.5
tpwr            63  rfp                0
pw           7.450  rp             -19.3
   DECOUPLER        lp            -102.5
dn             C13             PLOT
dof              0  wc               250
dm             nnn  sc                 0
dmm              c  vs               134
dpwr            46  th                 3
dmf          18500  ai   cdc   ph
```

INDEX	FREQUENCY	PPM	HEIGHT
1	3176.821	7.940	22.6
2	3112.389	7.779	21.1
3	3109.265	7.772	23.9
4	3106.922	7.766	25.8
5	3103.993	7.758	32.9
6	3099.698	7.748	6.5
7	3083.882	7.708	7.2
8	3079.587	7.697	36.5
9	3077.049	7.691	30.8
10	3074.510	7.685	26.1
11	3070.020	7.673	40.7
12	3059.867	7.648	27.5
13	3051.471	7.627	11.4
14	3028.822	7.570	46.5
15	2999.925	7.498	9.1
16	2994.067	7.484	78.4
17	2992.115	7.479	45.5
18	2987.819	7.468	41.7
19	2980.009	7.448	11.3
20	2976.104	7.439	3.8
21	2972.590	7.430	8.4
22	2970.247	7.424	12.0
23	2967.513	7.417	9.8
24	2964.194	7.409	10.7
25	2960.289	7.399	5.1
26	2957.946	7.393	4.5
27	2903.471	7.257	10.4
28	1706.979	4.267	14.2
29	1699.950	4.249	25.1
30	1692.726	4.231	14.2
31	1491.423	3.728	3.8
32	1483.223	3.707	162.3
33	1477.756	3.694	88.3
34	1401.608	3.503	16.6
35	1394.970	3.487	34.3
36	1388.136	3.470	16.9
37	800.433	2.001	18.7
38	668.249	1.670	3.9
39	661.220	1.653	10.2

INDEX	FREQUENCY	PPM
40	653.801	1.634
41	646.576	1.616
42	638.962	1.597
43	599.716	1.499
44	592.883	1.482
45	585.073	1.462
46	577.848	1.444
47	571.015	1.427
48	499.944	1.250

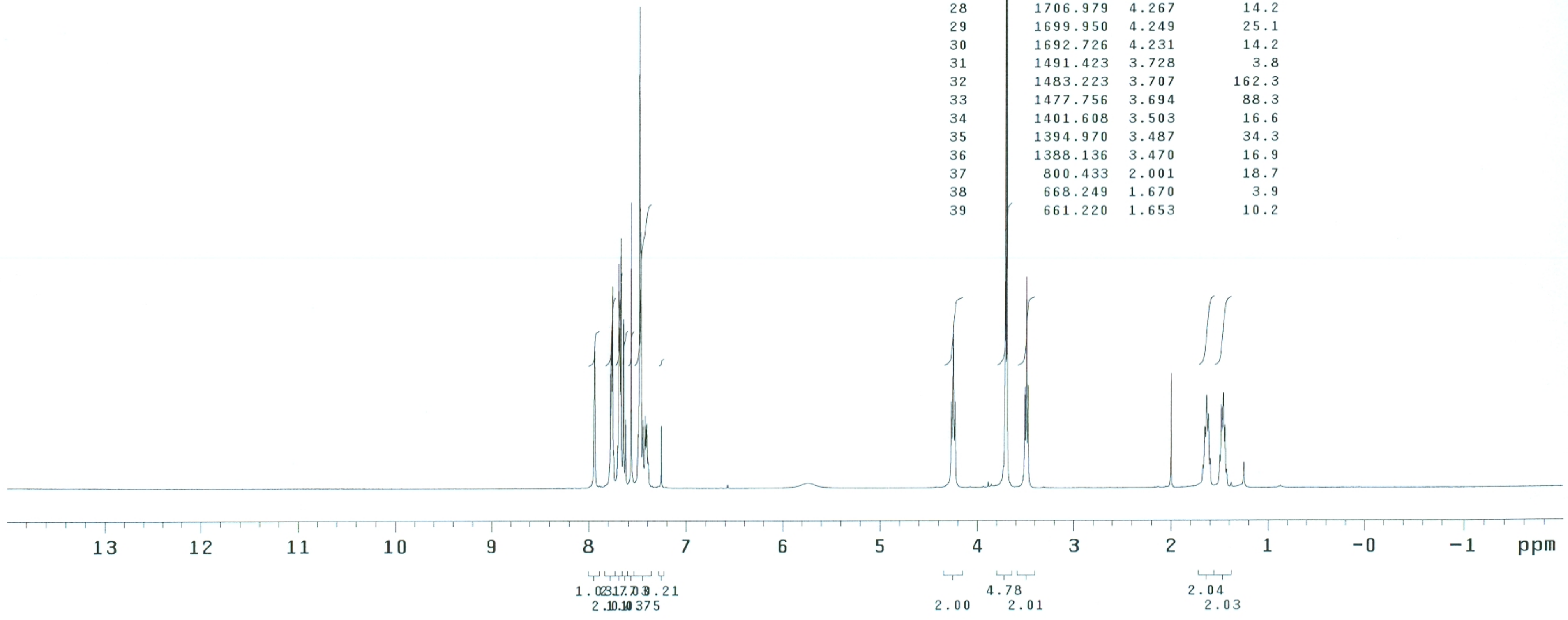

Supplement: Figure S1 — Proton NMR spectrum of 2. (0.79 MB PDF) [file pone.0010129.s001.pdf]
