# Supplementary figures and images for: Small Molecules Showing Significant Protection of Mice against Botulinum Neurotoxin Serotype A
Source: PLoS One. 2010 Apr 13;5(4):e10129. doi: 10.1371/journal.pone.0010129 (PMC2854131; doi:10.1371/journal.pone.0010129)

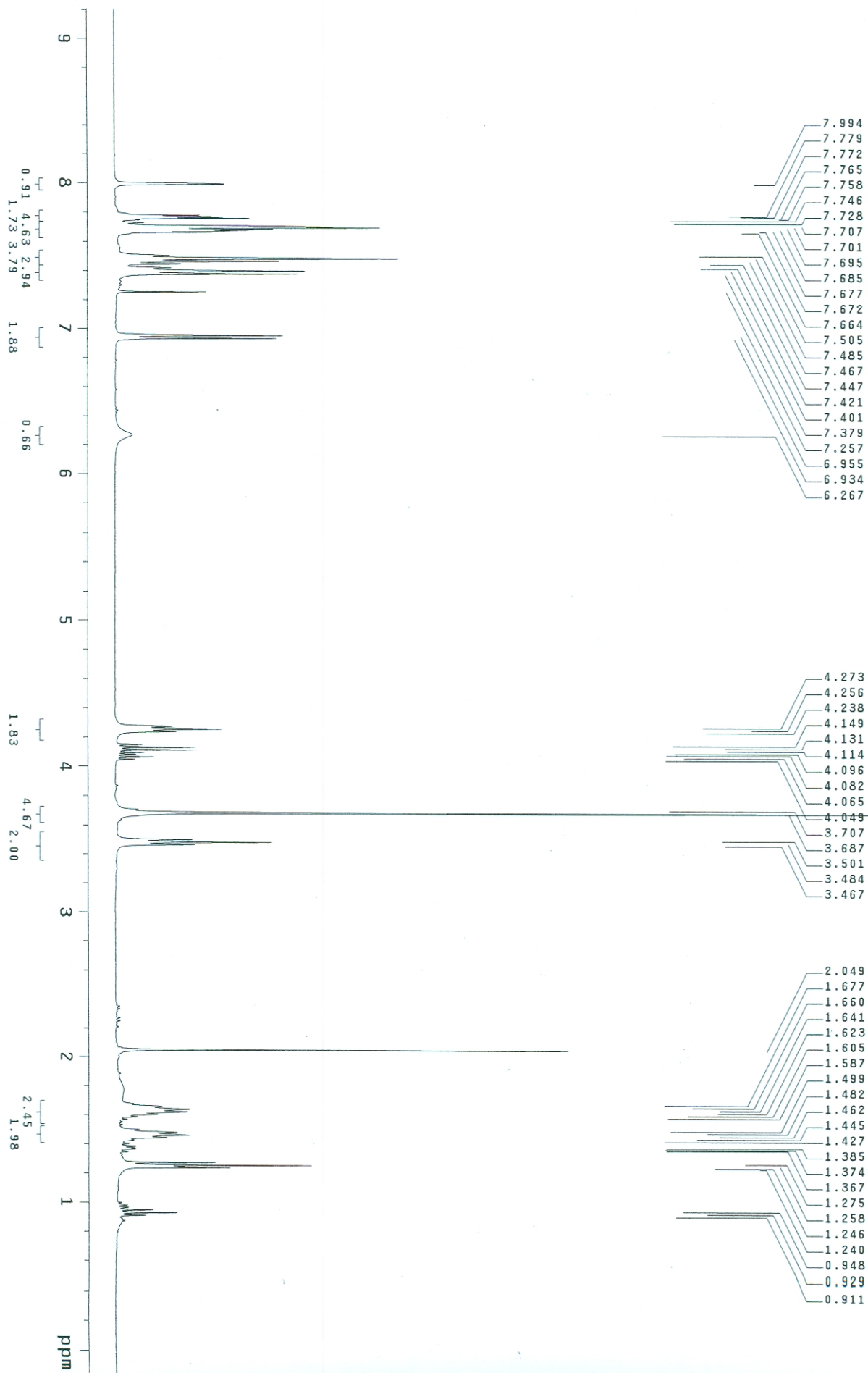

Supplement: Figure S2 — Proton NMR spectrum of 3x. (0.56 MB PDF) [file pone.0010129.s002.pdf]

# F4H before HPLC

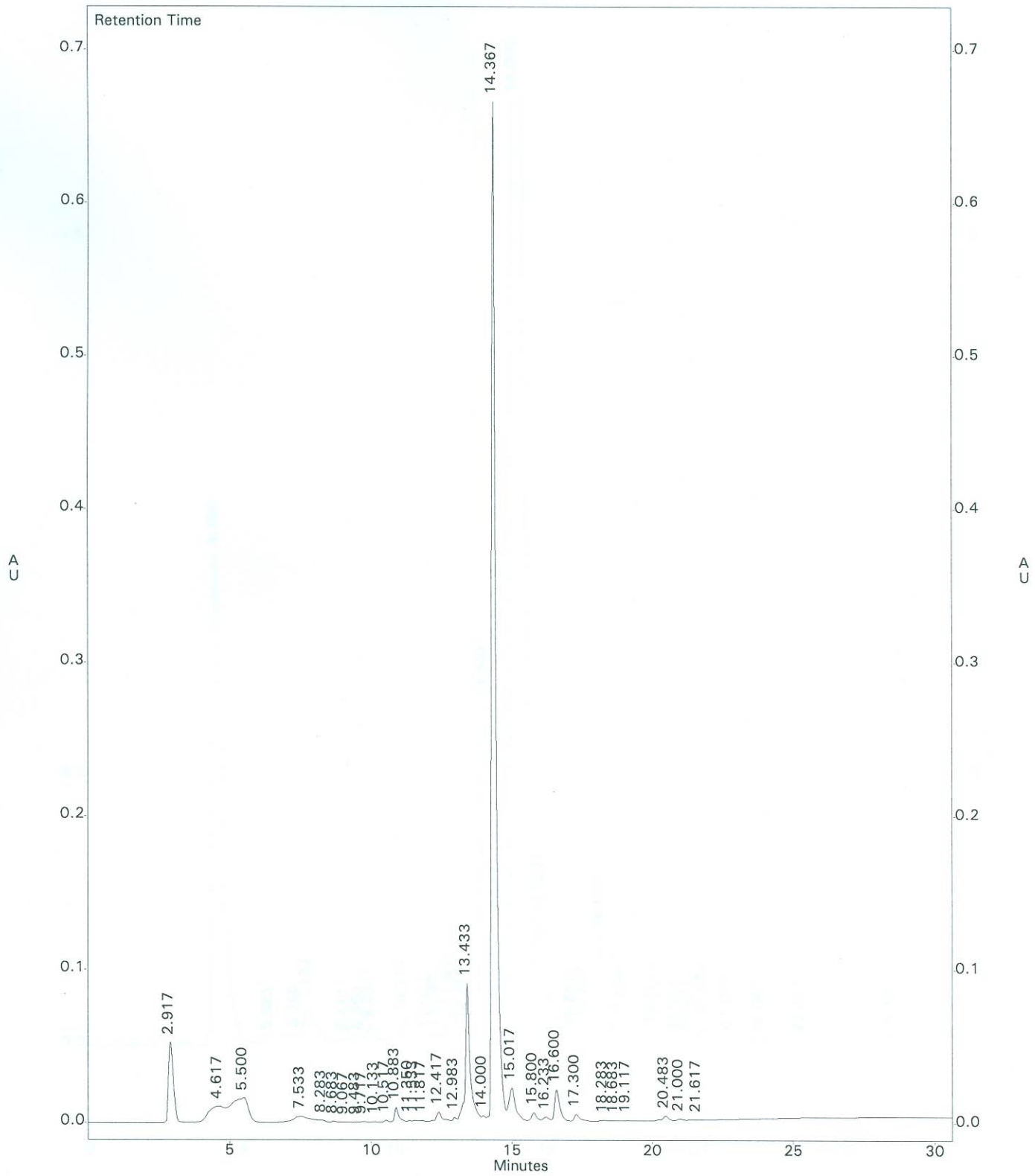

# F4H after HPLC

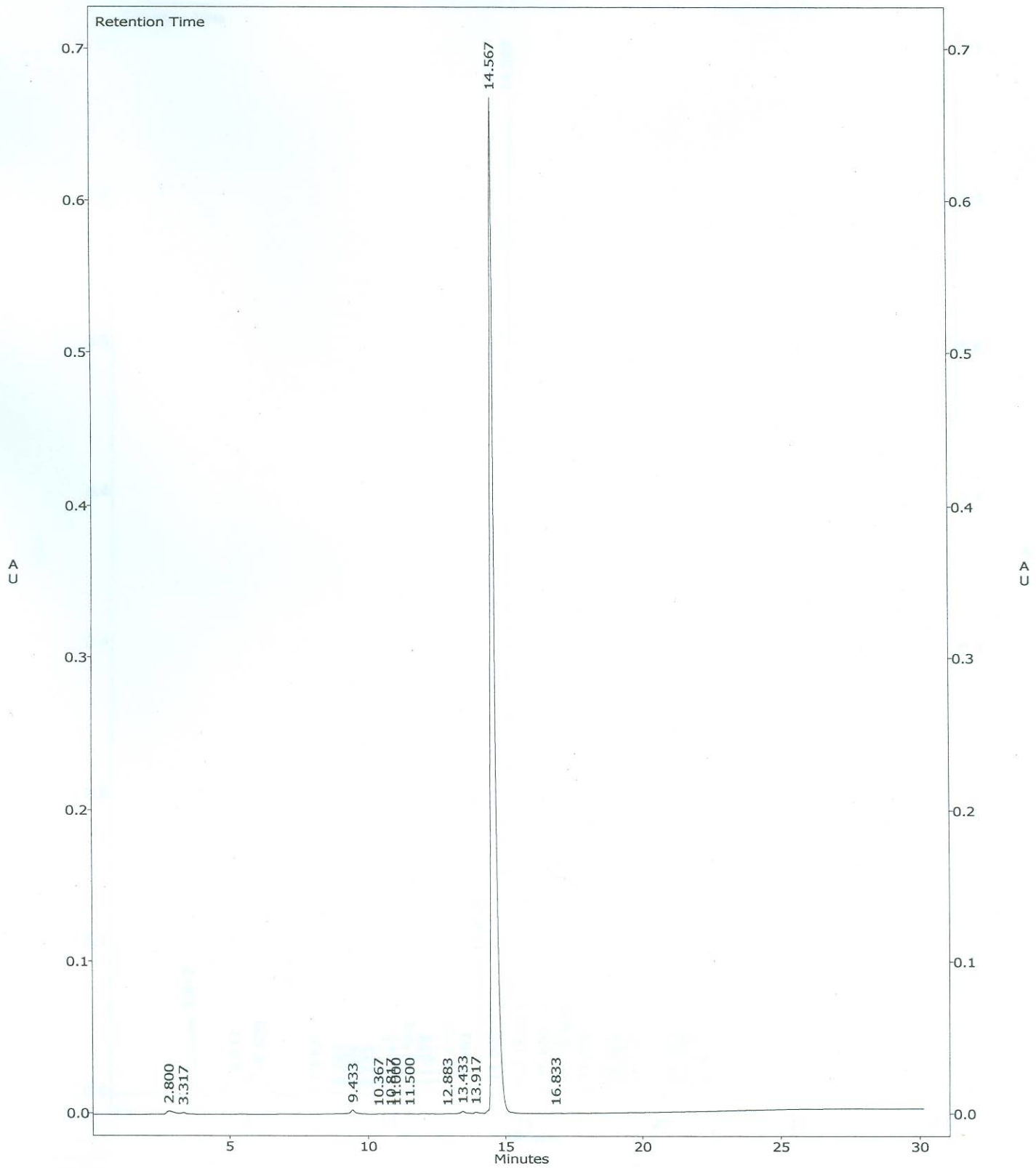

Supplement: Figure S3 — Chromatograms of F4H⋅TFA before and after the HPLC purification. (0.17 MB PDF) [file pone.0010129.s003.pdf]

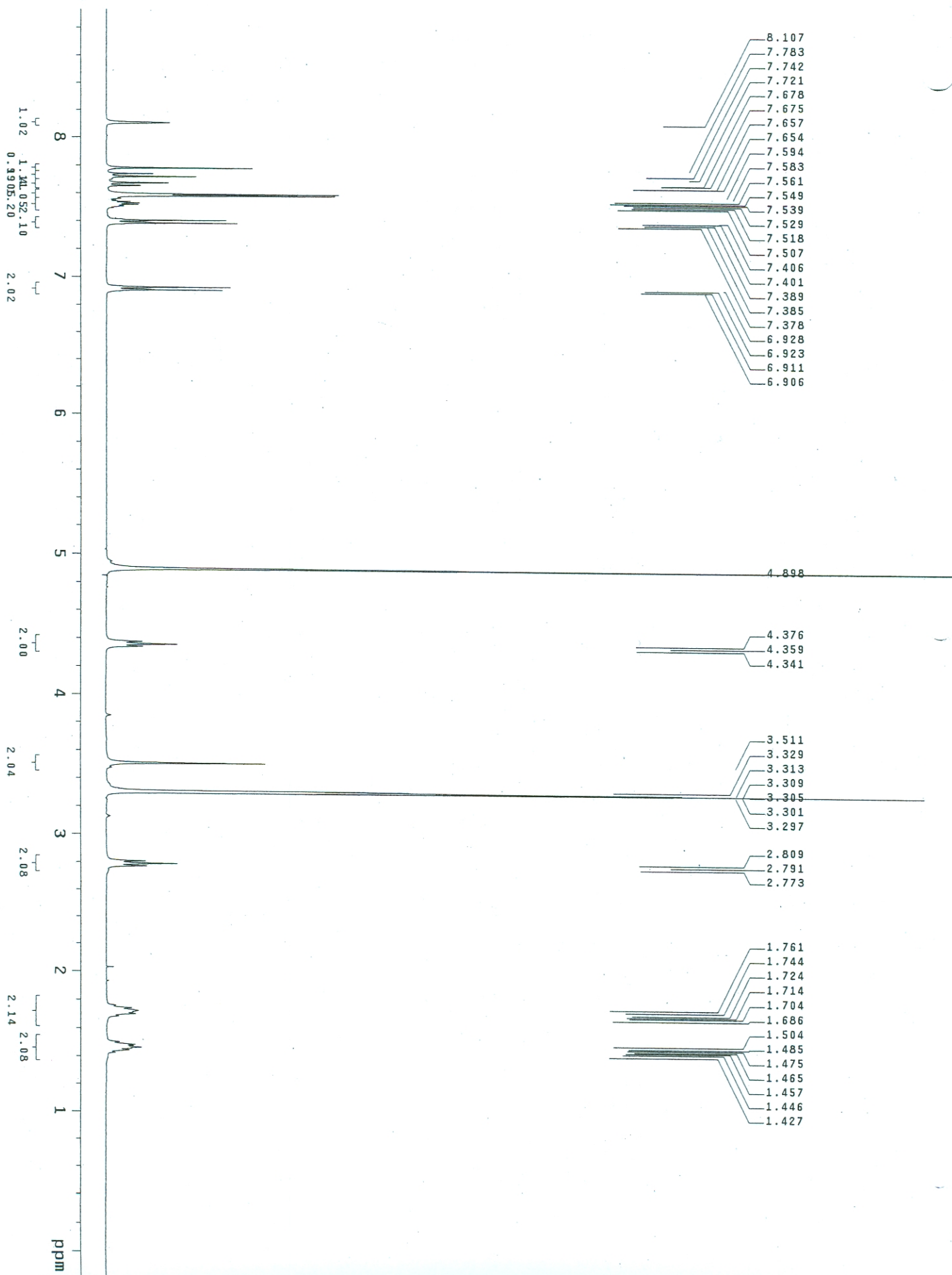

Supplement: Figure S4 — Proton NMR spectrum of F4H. (0.54 MB PDF) [file pone.0010129.s004.pdf]

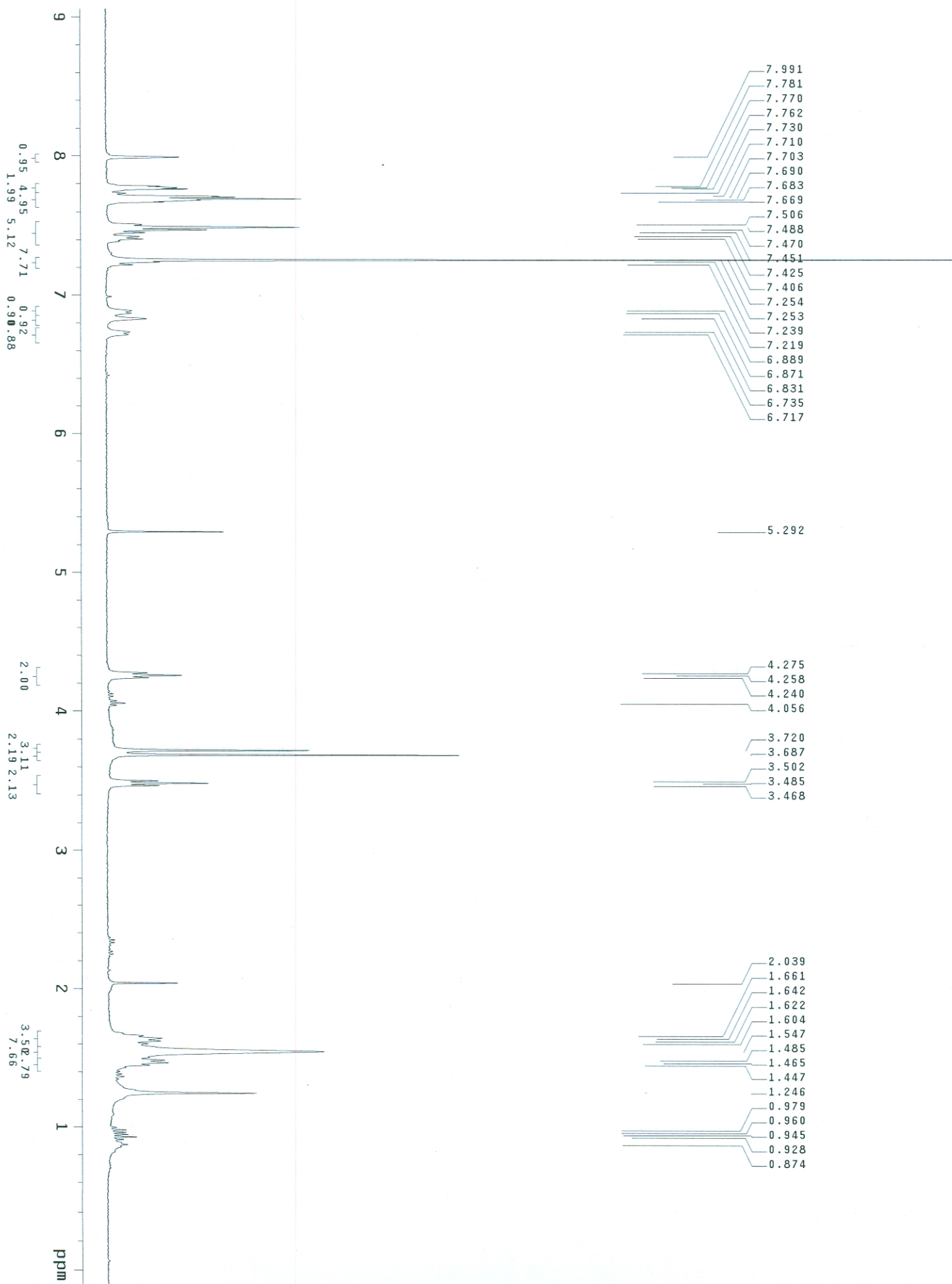

Supplement: Figure S5 — Proton NMR spectrum of 3y. (0.50 MB PDF) [file pone.0010129.s005.pdf]

# F3A before HPLC

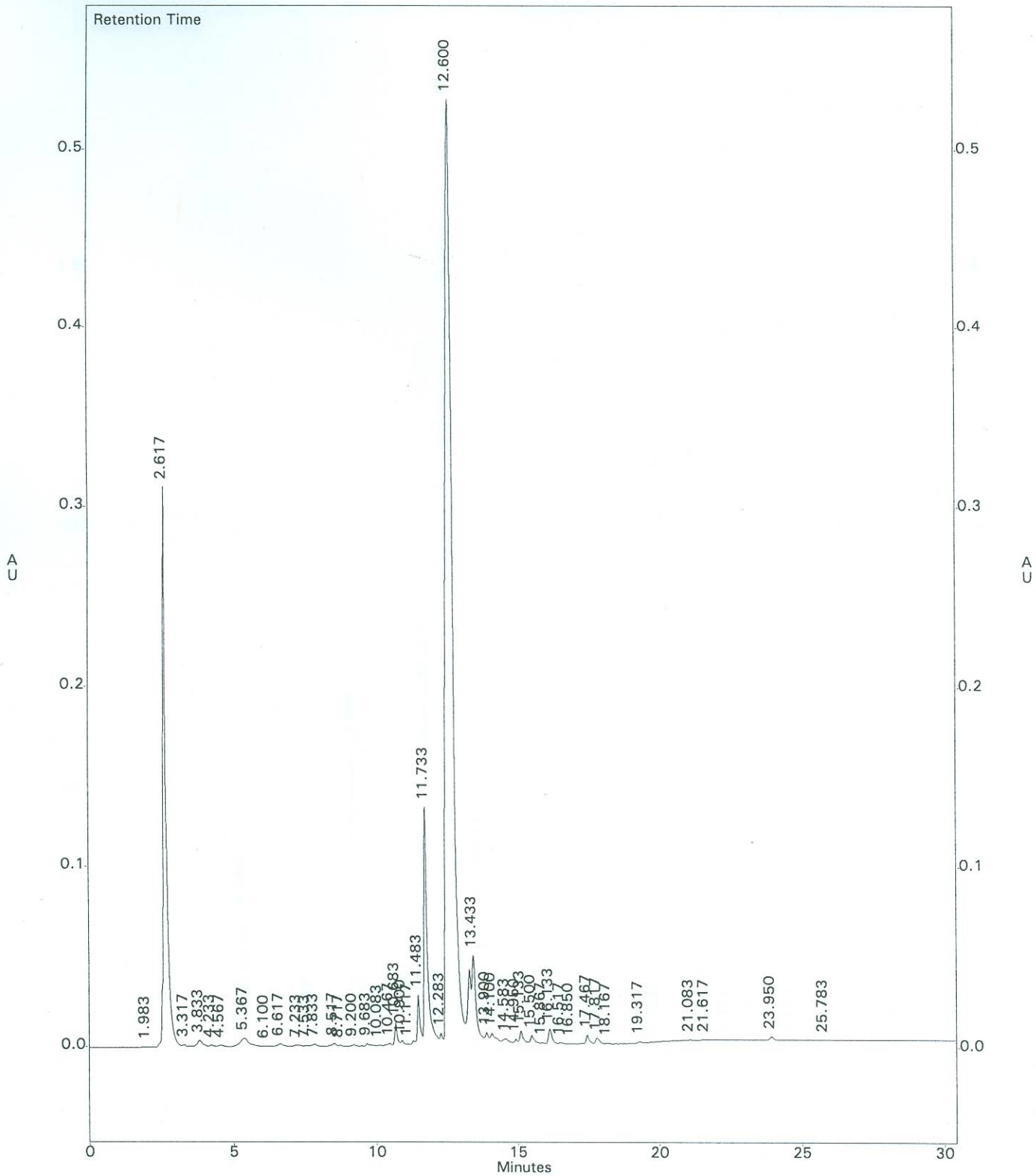

# F3A after HPLC

Retention Time

A  
U

1.0

0.5

0.0

A  
U

1.0

0.5

0.0

12.250

2.950

3.850

6.783

8.600

9.067

9.783

10.217

11.133

11.417

11.750

Minutes

0

5

10

15

20

25

30

Supplement: Figure S6 — Chromatograms of F3A⋅2TFA before and after the HPLC purification. (0.17 MB PDF) [file pone.0010129.s006.pdf]

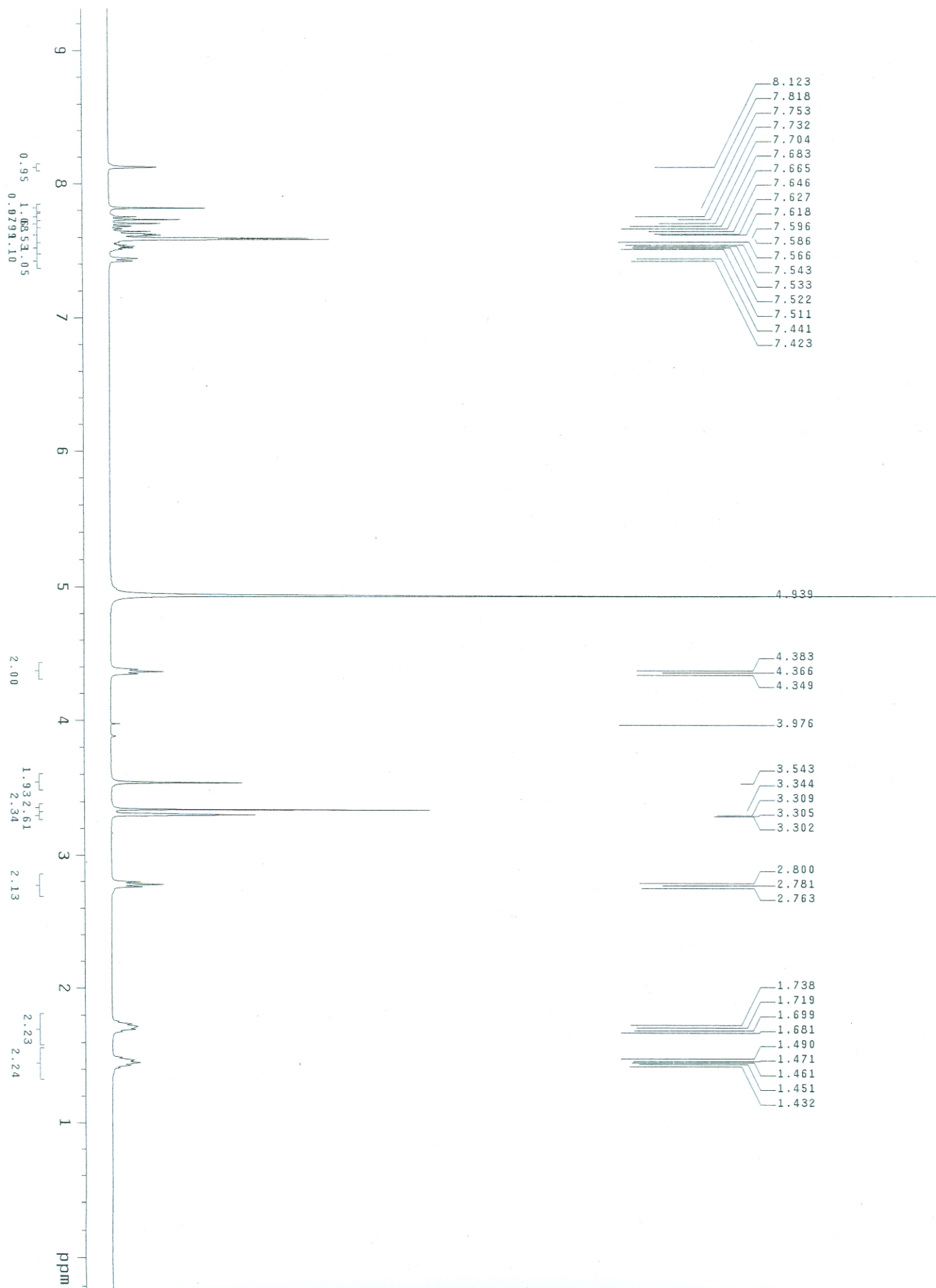

Supplement: Figure S7 — Proton NMR spectrum of F3A. (0.43 MB PDF) [file pone.0010129.s007.pdf]
